# Advancing breast cancer diagnosis: a combined approach using deep learning-reconstructed diffusion-weighted imaging and Synthetic MRI

**DOI:** 10.3389/fonc.2026.1777318

**Published:** 2026-07-15

**Authors:** Wanjun Xia, Qichang Fu, Kaiyu Wang, Lin Li, Anning Sai, Yong Zhang

**Affiliations:** 1 Department of Magnetic Resonance, The First Affiliated Hospital of Zhengzhou University, Zhengzhou, China; 2 MR Research China, GE Healthcare, Beijing, China; 3 Department of Breast Surgery, The First Affiliated Hospital of Zhengzhou University, Zhengzhou, China

**Keywords:** breast cancer, deep learning reconstruction, differential diagnosis, diffusion-weighted imaging, synthetic MRI

## Abstract

**Background:**

Breast cancer is the most common cause of malignancy in women, excluding skin cancer, leading to increased interest in novel non-invasive imaging techniques that eliminate the need for contrast agents.

**Objective:**

This study aims to establish a deep learning-reconstructed diffusion-weighted imaging (DL-DWI) model combined with Synthetic MRI and to evaluate its diagnostic value for breast cancer in this proof of concept study.

**Methods:**

A total of 111 patients with pathologically confirmed breast lesions (39 benign, 72 malignant) were enrolled, and all patients underwent DL-DWI and synthetic MRI scans. Quantitative parameters including ADC, DL-ADC, T1, T2 and PD were compared between benign and malignant groups using rank sum tests. ROC curve analysis was used to assess the diagnostic efficacy of single and combined models, and DeLong’s test was applied for pairwise AUC comparison.

**Results:**

Both DL-ADC (AUC = 0.993; 95% CI: 0.955-1.000) and conventional ADC (AUC = 0.990; 95% CI: 0.949-1.000) demonstrated excellent and comparable diagnostic accuracy. The DL-ADC derived from DL-DWI demonstrated excellent diagnostic accuracy (AUC = 0.993), comparable to conventional ADC (AUC = 0.990). A model combining Synthetic MRI with DL-DWI yielded an AUC of 0.995. However, DeLong’s test revealed no statistically significant differences in AUC between any pair of models (all p > 0.05): Synthetic MRI + DL-DWI vs. DL-DWI alone (p=0.45), Synthetic MRI + DL-DWI vs. DWI alone (p=0.54), and DL-DWI vs. DWI alone (p=0.67).

**Conclusion:**

In this proof of concept study, standalone DL-DWI achieves high accuracy in differentiating benign and malignant in breast lesions meeting the study size criteria. Adding synthetic MRI brings no significant diagnostic improvement, indicating DL-ADC is the dominant diagnostic biomarker. This contrast-free deep learning-based MRI approach has potential as a contrast free protocol and should be further evaluated in a larger clinical trial comparing contrast-enhanced breast MRI with this novel technique.

## Highlights

- DL-DWI alone achieved excellent diagnostic performance (AUC: 0.993) for differentiating breast lesions.
- This approach presents a promising alternative that could potentially reduce reliance on contrast-enhanced MRI.
- DL reconstruction improves performance via clearer images and better measurements than conventional methods.

## Introduction

Breast cancer is the most common cause of malignancy in women worldwide excluding skin cancer. Breast magnetic resonance imaging is an advanced imaging technique with high sensitivity and specificity (1, 2), however there is room for improvement (3, 4).

Breast lesion screening is critical for women’s health. Despite reliable diagnostic performance, conventional breast MRI suffers from a high false-positive rate, leading to unnecessary invasive pathological biopsies for final diagnosis. Moreover, routine contrast-enhanced breast MRI relies on exogenous contrast agents, which may cause gadolinium deposition and are contraindicated for patients with renal insufficiency. Therefore, non-contrast breast MRI has become a hotspot of clinical research (5, 6).

Quantitative synthetic MRI (SyMRI) is a mature commercial multi-parametric imaging technique applied in clinical practice (7). It can acquire T1, T2 and proton density (PD) quantitative maps via a single scan, which reflect tissue water content, fat composition and microstructural features. Different conventional weighted images can be further generated based on these quantitative maps. Distinct from conventional synthetic imaging application, this study directly adopts quantitative SyMRI parameters for breast tissue characterization, which improves differential diagnosis efficiency of breast lesions (8, 9). Existing studies have verified that SyMRI quantitative parameters can reflect microstructural variations of breast lesions and effectively differentiate benign and malignant lesions (10, 11).

Diffusion-weighted imaging (DWI) is a routine sequence for breast examination. Deep learning reconstructed DWI (DL-DWI) can optimize image quality by improving signal-to-noise ratio and reducing imaging artifacts (12, 13). The goal of this study is to combine contrast-free SyMRI and DL-DWI to study its potential for non contrast breast screening and reduce unnecessary biopsies, bringing more benefits to patients.

## Methods

### Participants

The trial was approved by a local institutional review board and was conducted as a prospective study. Written informed consent was obtained from all subjects in this study. From January to October 2023, female patients with breast lesions who underwent MRI examinations at our hospital. Pathological results were obtained, with a maximum time difference of one week between the MRI and pathological examinations. A total of 111 cases were included, divided into 39 benign and 72 malignant lesion groups based on the pathological results. Patients with incomplete clinical data or unclear, low-quality images were excluded from the study. The breast MRI examinations were performed as part of the diagnostic work-up for patients with suspicious findings identified on prior imaging (mammography and/or ultrasound) or for preoperative staging in patients with a newly diagnosed breast cancer. Critically, no lesions occult on conventional mammography/ultrasound and only detectable on contrast-enhanced MRI (CE-MRI) were included in this proof-of-concept study. Specifically, indications included: further characterization of a BI-RADS 4 or 5 lesion found on mammography or ultrasound, determination of the extent of disease (multifocality/multicentricity) and contralateral breast involvement in patients with a biopsy-proven malignancy, and evaluation of response to neoadjuvant chemotherapy in a subset of cases.

As part of the inclusion criteria, all lesions included in the study met size requirements to ensure valid quantitative measurement. For solid space-occupying lesions (accounting for the majority of benign and malignant lesions in the cohort), the diameter was required to be greater than 1 cm -– this threshold was sufficient to visualize the lesion on at least three consecutive 5 mm-thick slices, allowing for stable ROI placement and quantitative parameter measurement. The 39 benign lesions included fibroadenomas, adenosis lesions, intraductal papillomas, adenosis with fibroadenomas, adenosis with duct dilatation, inflammation, and adenomyoepitheliomas; the 72 malignant lesions included invasive carcinoma, intraductal papillary carcinoma, ductal carcinoma *in situ* (DCIS), invasive carcinoma with DCIS, lobular carcinoma *in situ* (LCIS), and lymph node metastasis. Notably, DCIS, LCIS, and a small portion of adenosis lesions presented as patchy, multiple, and ill-defined lesions without obvious solid masses, making accurate three-dimensional size (length, width, height) measurement impractical. For these three types of lesions, the inclusion criterion was adjusted to require the long diameter of the largest single lesion to be greater than 1 cm, ensuring the lesion could be identified on three consecutive slices for ROI measurement.

All MRI scans were performed prior to surgical intervention, definitive biopsy, or any treatment including neoadjuvant chemotherapy.

### MRI acquisition

MRI examinations were performed using a 3T GE scanner (SIGNATM Premier, GE Healthcare, Waukesha, WI) with an 8-channel breast phased array surface coil. Both breasts were naturally suspended in the center of the coil. A total of 111 female patients with breast lesions underwent routine DWI, DL-DWI, and Synthetic MRI scans. The DWI sequence parameters were as follows: b value = 800 s/mm^2^, TR/TE = 2500.03 ms/54.3 ms, thickness = 5 mm, FA = 90°, FOV = 340 mm × 340 mm, matrix = 128 × 128, with a conventional protocol NEX = 6 and DL protocol NEX = 4. The conventional DWI scan time was 2 minutes and 28 seconds, while the DL-DWI scan time was 2 minutes. Contrast enhanced MR images were collected but not part of this analysis and reserved for a future study.

The DL-DWI images were reconstructed using a commercially available, vendor-provided deep learning pipeline (AIR™ Recon DL, version SIGNA-LX1.MR30.1-R01B-2430.C, GE Healthcare) implemented directly on the scanner’s reconstruction system. This is a proprietary, FDA-cleared algorithm trained by the manufacturer to reduce noise and artifacts while preserving diagnostic features. As the model’s architecture and training parameters are not publicly disclosed, we utilized the product’s highest available noise reduction setting (‘High’). Similarly, the Synthetic MRI quantitative maps (T1, T2, and PD) were generated using the vendor’s integrated, quantitative MR post-processing suite (MAGiC, version SIGNA-LX1.MR30.1-R01B-2430.C, GE Healthcare), which is based on the methodology described by Hagiwara et al. (7). The scanning parameters for the Synthetic MRI sequence were: TR = 7000 ms, TE = 16.24 ms, FA = 90°, and layer thickness = 5 mm. The selected slice thickness of 5 mm represents a standard clinical compromise between acquisition time, volumetric coverage, and signal-to-noise ratio for both the DWI and Synthetic MRI sequences in a breast imaging protocol. While higher spatial resolution is desirable to minimize partial volume effects, the current parameters were chosen to ensure robust quantitative map generation and clinical feasibility within a reasonable scan time. It should be noted that the DL-DWI protocol utilized a reduced number of excitations (NEX = 4) compared to the conventional DWI (NEX = 6), contributing to its shorter scan time. Therefore, the observed improvements in the DL-DWI protocol represent the combined effect of the proprietary deep learning reconstruction and the acquisition parameter adjustment, rather than the effect of the reconstruction algorithm alone.

### MRI interpretation

Data processing was conducted independently by three diagnostic MRI practitioners with 16, 6, and 1 year of experience, respectively. For each lesion, the region of interest (ROI) was placed on the solid portion of the lesion as visualized on the high-resolution, synthesized T2-weighted image generated by the Synthetic MRI sequence. This ROI was then automatically propagated onto the co-registered quantitative maps (T1, T2, PD) and the apparent diffusion coefficient (ADC)/DL-ADC maps using the workstation’s software. Care was taken to avoid areas of cystic change, liquefaction, necrosis, and hemorrhage. Notably, all pathologically confirmed lesions included in this study were clearly identifiable on routine clinical imaging (mammography, ultrasound, and conventional breast MRI); no lesions were excluded due to poor visibility on synthesized T2-weighted images, thus eliminating potential selection bias. Each quantitative parameter was measured three times across consecutive slices, and the average value was calculated. The final value used for statistical analysis for each patient was derived from a consensus review, where any initial measurement discrepancies greater than 10% were re-evaluated jointly to reach an agreed-upon ROI placement and measurement. Notably, this expert-driven, idealized ROI sampling strategy represents an idealized research workflow, which does not fully reflect the lesion heterogeneity and variable operation modes in routine clinical practice.

### Construction of the combined diagnostic model

A binary logistic regression model was initially designed as a combined multi-parameter model to differentiate malignant from benign pathology. Predictors included Z-score standardized DL-ADC, T1, and PD values. After variable selection, only standardized DL-ADC remained a significant independent predictor. The final model was defined as:

Logit(P) = -4.776 + 8.968 × (Z-scored DL-ADC), where P is the probability of malignancy.

### Statistical analysis

Data analysis and plotting were performed using SAS 9.4 (https://www.sas.com/zh_cn/home.html) and MedCalc (https://www.medcalc.org/). We perform a paired Wilcoxon signed-rank test) comparing the DL-ADC and conventional ADC values.This is the most appropriate test to determine if the two measurement methods yield significantly different values from the same tissue.The rank sum test was used to analyze differences in quantitative parameters (including ADC, DL-ADC, T1, T2, and PD) between benign and malignant breast lesions. A *p*-value of < 0.05 was considered statistically significant. The diagnostic performance of the parameters was expressed using the Receiver Operating Characteristic (ROC) curve. Pairwise comparisons of ROC curves between models were performed using DeLong’s test. A Bonferroni correction was applied to account for multiple comparisons, setting the significance threshold at p < 0.0167 for the three tests performed.

## Results

### Participants

A total of 111 female patients, aged 51 ± 10 years, were studied. The cohort included 39 cases in the benign lesion group and 72 cases in the malignant lesion group, with 53 lesions in the left breast and 58 in the right breast, as shown in **Table 1**.

**Table 1:** Clinicopathologic characteristics of 111 breast lesions of women.

Characteristic	Value
Age (y)	51 ± 10
Benign lesions (39 lesions)	
Fibroadenoma	14(35.9)
adenosis	2(5.1)
intraductal papilloma	2(5.1)
Adenopathy with a fibroadenoma	6(15.4)
Adenopathy was associated with ductal dilatation	10(25.6)
inflammation	4(10.3)
adenomyoepithelioma	1(2.6)
Malignant lesions (72 lesions)	
infiltrating cancer	50(69.4)
Intraductal papillary carcinoma	3(4.2)
ductal carcinoma in situ(DCIS)	1(1.4)
Invasive carcinoma with DCIS	6(8.3)
lobular carcinoma in situ	1(1.4)
Lymph node metastases	11(15.3)

Unless stated otherwise, data in parentheses are percentages. Percentages may not add up to 100 because of rounding.

### Comparison of conventional DWI and DL-DWI

**Figure 1:**
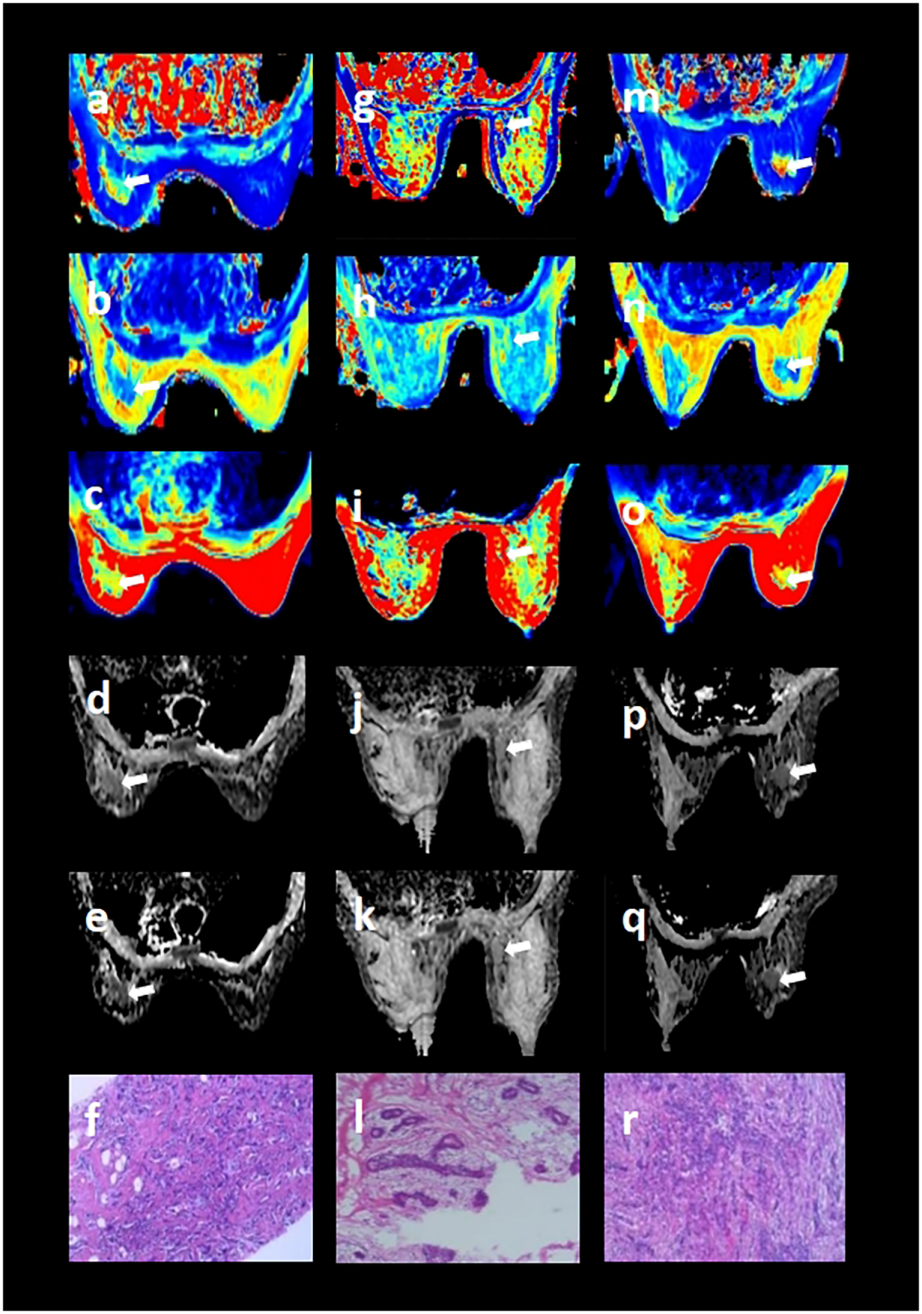
Parametric maps and pathological findings in breast lesions. White arrows indicate lesions. **(a–f)** 45-year-old patient with left breast invasive cancer: **(a)** T1 mapping, **(b)** T2 mapping, **(c)** PD mapping, **(d)** conventional ADC, **(e)** deep learning-reconstructed DWI–derived lesion ADC, **(f)** pathology. **(g–l)** 30-year-old patient with right breast fibroadenoma: **(g)** T1 mapping, **(h)** T2 mapping, **(i)** PD mapping, **(j)** conventional ADC, **(k)** deep learning-reconstructed DWI–derived lesion ADC, **(l)** pathology. **(m–r)** 45-year-old patient with right breast invasive cancer: **(m)** T1 mapping, **(n)** T2 mapping, **(o)** PD mapping, **(p)** conventional ADC, **(q)** deep learning-reconstructed DWI–derived lesion ADC, **(r)** pathology.

**Figure 2:**
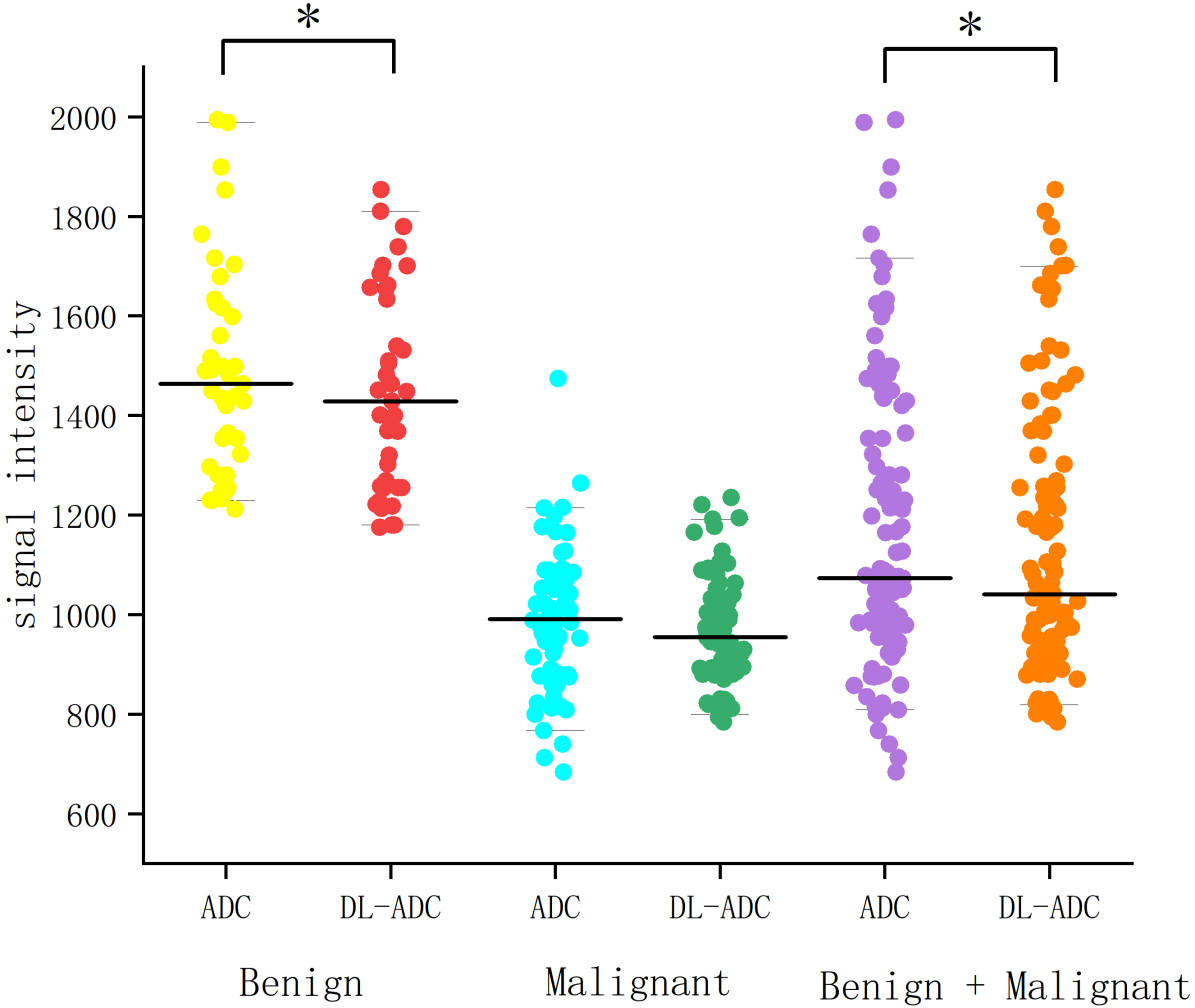
Scatter plot comparison of ADC and DL−ADC values. Comparison between conventional ADC and DL−ADC values in benign lesions, malignant lesions, and the overall cohort. A significant difference was observed in the benign group (p = 0.0279) and across all cases (p = 0.0085), whereas no significant difference was found in the malignant group (p = 0.1158). Asterisk (*) indicates a statistically significant difference.

The DWI scan for deep learning reconstruction (2 minutes) is faster than the traditional DWI scan (2 minutes 28 seconds). After deep learning, the ADC values in the benign lesion group were significantly different from those in the malignant lesion group, with the benign lesions showing higher ADC values (*p* < 0.05), as illustrated in **Table 2** and **Figure 1**. Additionally, the ADC values obtained after deep learning reconstruction were lower than the conventional ADC values (p = 0.0085, 95%CI -42.4500 - -6.7500), as shown in **Figure 2**.

**Table 2:** Comparison of parameters from DWI and synthetic MRI between the two groups.

Item	B800 (10^-3^mm^2^/s)	DL800 (10^-3^mm^2^/s)	T1 (ms)	T2 (ms)	PD (pu)
Malignant group(n=72)	0.994 ± 0.136	0.971 ± 0.109	1928.3 ± 334.767	97 ± 22.327	82.41 ± 17.64
Benign group(n=39)	1.491 ± 0.215	1.448 ± 0.201	1564 ± 476.652	99 ± 14.863	106 ± 17.008
p	<0.0001	<0.0001	<0.0001	0.1029	<0.0001
95% CI	-0.625 - -0.417	-0.570 - -0.382	57.5 - 484	-16 - 3	-26.8- -14.8

B800 s/mm^2^ represents ADC derived from conventional reconstructed DWI at b of 800 s/mm^2^; DL800 represents ADC derived from deep learning-reconstructed DWI at b of 800 s/mm^2^.

**Figure 3:**
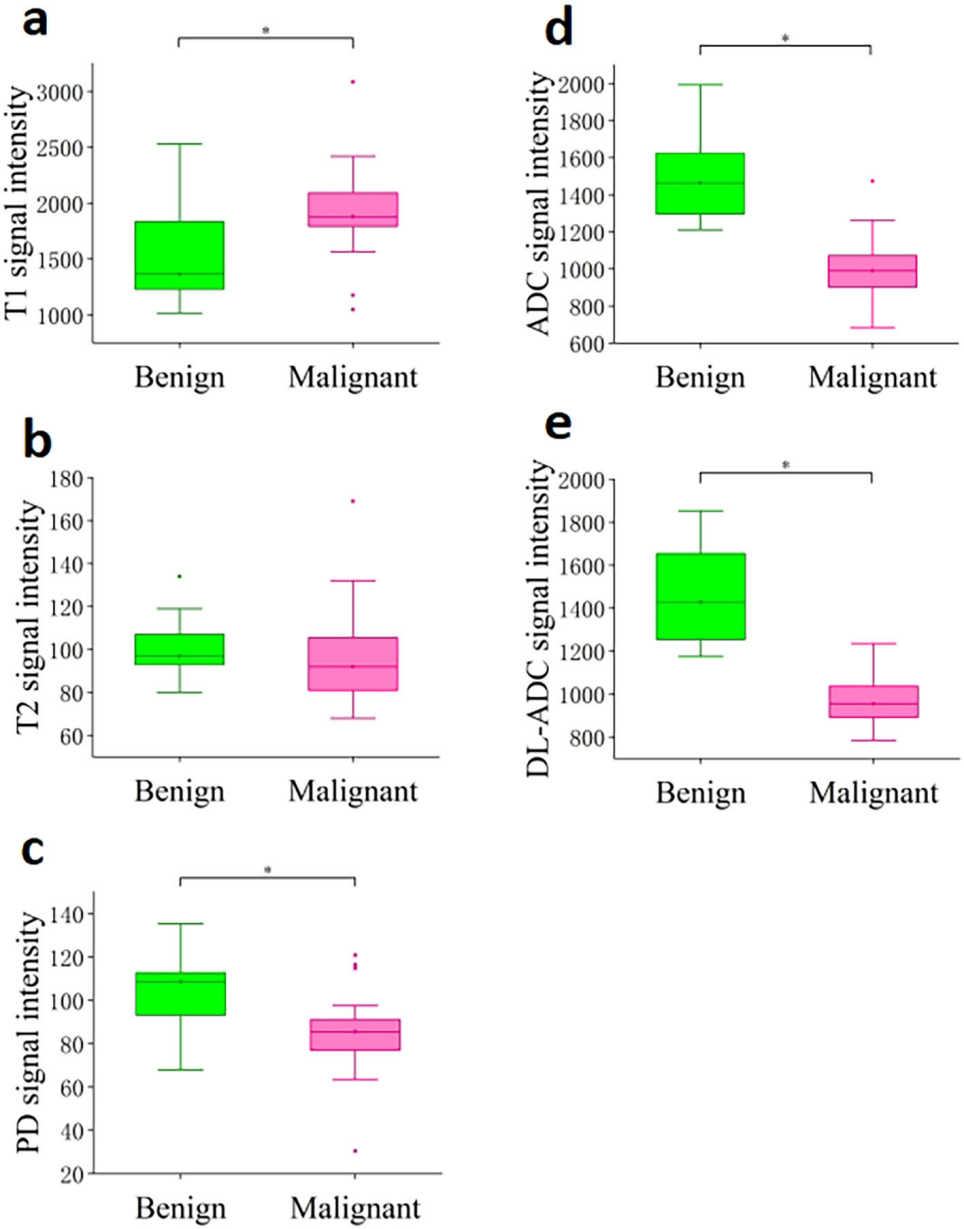
Box plots comparing T1 **(a)**, T2 **(b)**, PD **(c)**, ADC **(d)**, and DL-ADC **(e)** values between benign and malignant groups. Asterisk (*) indicates a statistically significant difference.

The DL-DWI protocol, employing both a reduced NEX and DL reconstruction, was faster (2 minutes) than the conventional DWI scan (2 minutes 28 seconds). The ADC values derived from the DL-DWI protocol (DL-ADC) in the benign lesion group were significantly higher than those in the malignant lesion group (p < 0.05) as shown in **Figure 3**. Furthermore, the mean DL-ADC values were lower than the conventional ADC values.

### Quantitative parameter analysis of synthetic MRI

There was no significant difference in T2 mapping between benign and malignant lesions (*p* > 0.05). The T1 mapping of malignant lesions was 1928.3 ms, significantly higher than that of benign lesions (*p* < 0.05). The PD mapping of malignant lesions was 82.41 pu, significantly lower than the 106 pu observed in benign lesions (*p* < 0.05), as shown in **Figures 1**, **3** and **Table 2**.

### ROC study of all parameters

**Figure 4:**
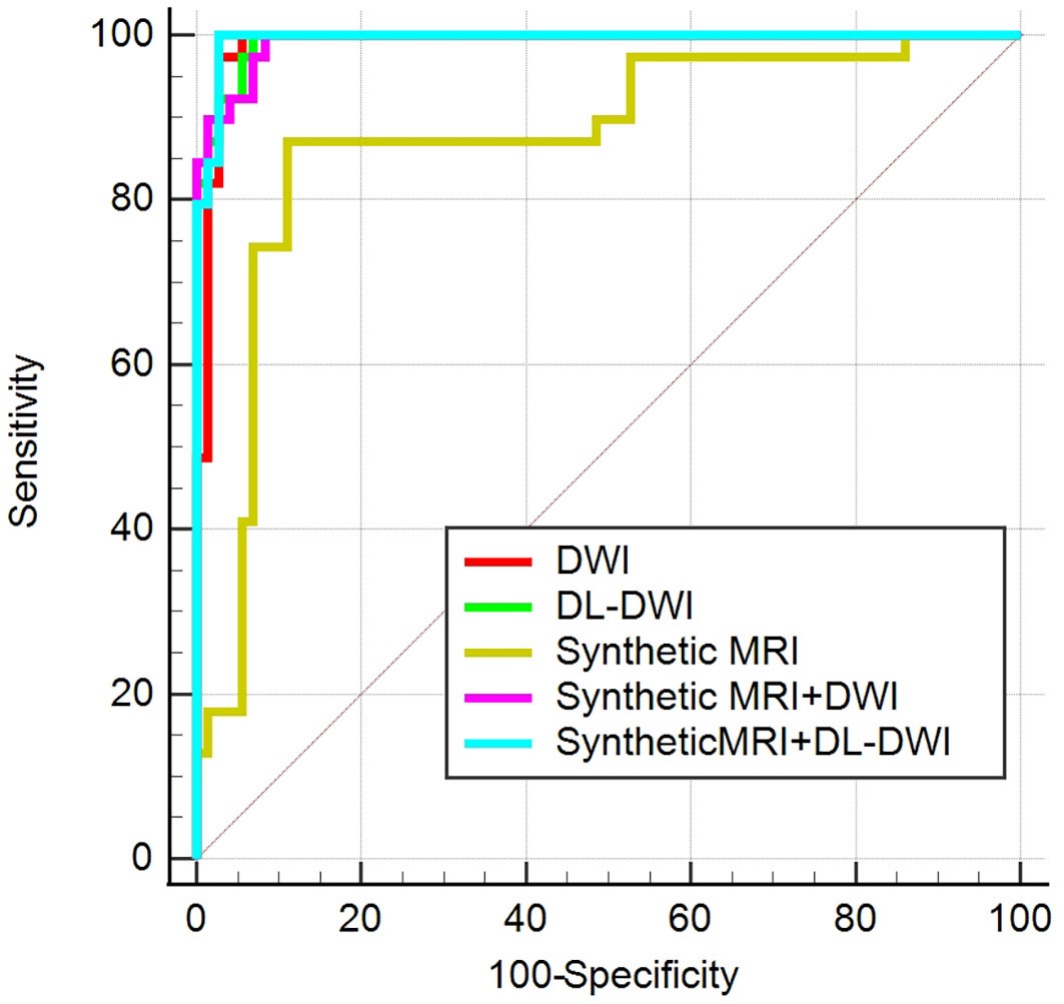
Receiver operating characteristic (ROC) curves for different models in differentiating benign and malignant breast lesions. The corresponding area under the curve (AUC) values are: Synthetic MRI + DL-DWI (AUC = 0.995), DL-DWI alone (AUC = 0.993), Synthetic MRI + DWI (AUC = 0.993), DWI alone (AUC = 0.990), and Synthetic MRI alone (AUC = 0.874).

The diagnostic performance of each parameter and model for differentiating benign from malignant lesions was evaluated using ROC analysis (**Table 3**; **Figure 4**). All individual parameters and models demonstrated AUCs significantly greater than 0.5 (all p < 0.0001). The DL-ADC value with cut-off value of 1.166 × 10–3 mm^2^/s from the DL-DWI model yielded excellent diagnostic accuracy (AUC = 0.993; 95% CI: 0.955–1.000), comparable to that of the conventional ADC value (AUC = 0.990; 95% CI: 0.949–1.000). The combination of Synthetic MRI parameters (T1 and PD mapping) resulted in a lower AUC of 0.874 (95% CI: 0.798–0.929), which was significantly inferior to the DL-ADC value (p < 0.05). The combined model integrating Synthetic MRI with DL-DWI achieved AUC of 0.995 (95% CI: 0.958–1.000), with a sensitivity of 100% and a specificity of 97.22%. Pairwise comparisons of the ROC curves using DeLong’s test, with a Bonferroni-corrected significance threshold of p < 0.0167, confirmed no statistically significant differences in AUC between any pair of models: Synthetic MRI + DL-DWI vs. DL-DWI alone (p = 0.45), Synthetic MRI + DL-DWI vs. DWI alone (p = 0.54), and DL-DWI vs. DWI alone (p = 0.67).

**Table 3:** ROC analyses of DWI and synthetic MRI parameters for differentiating benign and malignant breast lesions.

Sequence	AUC	95% CI	p	Sensitivity (%)	Specificity (%)
DWI	0.990	0.949 - 1.000	<0.0001	97.44	97.22
DLDWI	0.993	0.955 - 1.000	<0.0001	100.00	93.06
T1 mapping	0.746	0.655 - 0.824	<0.0001	71.79	93.06
PD mapping	0.834	0.752 - 0.898	<0.0001	82.05	81.94
Synthetic MRI	0.874	0.798 - 0.929	<0.0001	87.18	88.89
Synthetic MRI+DWI	0.993	0.953 - 1.000	<0.0001	100.00	91.67
Synthetic MRI+DLDWI	0.995	0.958 - 1.000	<0.0001	100.00	97.22

The p-value for each row corresponds to the test of the null hypothesis that the AUC equals 0.5. The “Synthetic MRI” model is based on a combination of the T1 and PD mapping parameters only, as T2 mapping did not show significant discriminatory power.

### *Post-hoc* statistical power analysis

A *post-hoc* statistical power analysis was performed to evaluate the adequacy of the current sample size, using dedicated statistical software for ROC curve comparison based on DeLong’s test. The primary study endpoint was the difference in AUC between different diagnostic models for distinguishing benign and malignant breast lesions. We set a two-sided type I error rate α=0.05. Given three pairwise comparisons of ROC curves in this study, the Bonferroni correction was applied, and the adjusted significance threshold was set to α=0.0167. With the observed sample sizes (39 benign lesions and 72 malignant lesions) and the actual AUC values acquired in this cohort, the analysis revealed that the current sample achieved sufficient statistical power (>80%) to detect moderate-to-large differences in diagnostic performance among the tested models.

Notably, the numerical differences in AUC across all models were extremely small (ranging from 0.990 to 0.995). The *post-hoc* power calculation confirmed that the present cohort lacked adequate power to identify such trivial differences, which is consistent with the non-significant results of DeLong’s test (all p>0.05). It should be emphasized that *post-hoc* power analysis has inherent limitations and cannot replace *a priori* sample size calculation performed before patient enrollment.

## Discussion

The ADC value, reflecting the molecular diffusion of water within tissue, has been well-established as a critical biomarker for characterizing breast lesions, with lower values typically indicating the restricted diffusion found in malignant tissues due to high cellularity (14–21). Our findings align with this principle, demonstrating that malignant lesions had significantly lower ADC values than benign ones.

The novel aspect of our study lies in the application of a deep learning-reconstructed DL-DWI. Paired analysis confirmed that DL-ADC values were significantly lower than conventional ADC values (p = 0.0085, 95% CI: = -42.4500 - -6.7500), supporting the hypothesis that DL reconstruction reduces noise bias and provides more accurate quantification. This observation is consistent with emerging literature suggesting that deep learning-based denoising reduces the noise floor, leading to more accurate and potentially lower ADC measurements (22–26). On the other hand, when a lower NEX acquisition is companied with a commercial deep learning reconstruction pipeline, this lower ADC cannot be disentangled from the concomitant change in acquisition parameters in our study design.

Our analysis of Synthetic MRI parameters yielded both expected and divergent results. While T2 mapping showed limited diagnostic value in our cohort, we found that malignant lesions exhibited significantly higher T1 values and lower PD values compared to benign lesions. The finding of reduced PD in malignancies is biologically plausible and supported by other studies; the high proliferation capacity of cancer cells reduces the extracellular space, thereby restricting water molecules and lowering PD (11).

However, our results highlight that the relationship between T1 and malignancy may be more complex than previously thought and could be subtype-dependent. Our finding that malignant lesions presented significantly higher T1 values than benign ones is inconsistent with several previous studies (27, 28). Several potential reasons may account for this discrepancy. First, breast cancer consists of diverse histological types and molecular subtypes, which vary greatly in cellular density, stromal components, necrosis and edema. The unique subtype distribution within our single-center cohort may lead to distinct T1 relaxation characteristics. Second, variations in MRI scanning parameters and vendor-specific Synthetic MRI reconstruction algorithms across different studies can also affect quantitative T1 measurements. Third, the proportion of heterogeneous components such as necrosis and inflammation in the enrolled lesions may further interfere with T1 values. It should be noted that all above interpretations are speculative, as we did not perform systematic correlation analysis between quantitative MRI parameters and detailed histopathological features, tumor grades or molecular biomarkers in the current study.

The diagnostic performance in our study was exceptionally high. The primary finding is that the DL-ADC value from the DL-DWI model alone yielded excellent diagnostic accuracy (AUC = 0.993), which was statistically equivalent to that of the conventional ADC (AUC = 0.990). Although a model combining Synthetic MRI parameters with DL-DWI achieved an AUC of 0.995, formal statistical comparison revealed it was not significantly superior to DL-DWI alone (p=0.45). This indicates that the DL-ADC parameter is the principal driver of the high diagnostic performance observed. Therefore, a streamlined protocol based on DL-DWI alone may offer a highly effective, rapid, and non-contrast diagnostic approach, with the addition of Synthetic MRI providing no statistically demonstrable gain in this cohort.

The exceptionally high AUCs reported in this study, while indicative of strong performance, must be contextualized within the study’s design, which may introduce optimistic bias. First, the cohort, though prospectively enrolled, was from a single institution and of limited size (n=111). This increases the risk of spectrum bias, where the included lesions (predominantly straightforward solid malignancies and benign fibroadenomas) may not represent the full spectrum of diagnostic challenges encountered in broader practice, such as non-mass enhancements or pure DCIS. Second, the manual placement of a single ROI on the ‘most solid portion’ of the lesion, guided by high-resolution synthetic T2 images, represents an idealized, expert-driven measurement scenario. In clinical practice, lesion heterogeneity, operator variability, and the presence of smaller or more diffuse lesions would introduce greater measurement variance, likely leading to more modest real-world performance. Consequently, the near-perfect accuracy observed here should be viewed as a demonstration of the technique’s potential under optimized, research-grade conditions rather than its expected performance in a routine clinical setting.

When we compare our results to the existing literature, the strengths of our deep learning-enhanced approach become apparent. Both the DL-DWI model and the combined model with Synthetic MRI achieved outstanding diagnostic accuracy (AUCs of 0.993 and 0.995, respectively). Historically, the benchmark for non-invasive diagnosis has often been DWI combined with Dynamic Contrast-Enhanced (DCE) MRI, which typically reports AUCs between 0.795 and 0.860 (29–31). While our non-contrast DL-DWI technique yielded numerically higher AUCs, a direct head-to-head comparison with DCE-MRI within the same patient cohort was not performed in this study. Therefore, claims of superiority or replacement are not supported by our present data. Instead, our key finding is the demonstration that deep learning-reconstructed DWI alone provides excellent diagnostic information. The fact that the multi-parametric combination with Synthetic MRI did not offer a statistically significant improvement over DL-DWI alone further suggests that the DL-ADC parameter is a primary driver of performance. This indicates that a streamlined, non-contrast protocol based on DL-DWI holds potential and merits future direct comparative evaluation against the standard DCE-MRI workflow to definitively assess its clinical role.

The near-perfect separation of groups, as evidenced by the high AUCs and the non-overlapping distributions in **Figure 4**, suggests that a clinically useful threshold for DL-ADC probably exists with the potential for both high sensitivity and high specificity. The exact threshold will require prospective validation in a larger, multi-center cohort to account for variations in scanners, populations, and disease prevalence.

Our study has several limitations that should be acknowledged. First, while we have provided the full logistic regression equation for the combined model, its development on a single-center cohort without internal cross-validation or an external test set risks overfitting. The extraordinarily high odds ratio warrants caution, and the model’s performance must be validated prospectively in independent, multi-center cohorts before clinical application can be considered. Second, the commercial deep learning and Synthetic MRI pipelines used are proprietary “black boxes.” While this ensures clinical applicability, it prevents a detailed technical understanding of the reconstruction process. Third, the comparison between conventional DWI and DL-DWI is confounded by differing acquisition parameters, specifically the reduced NEX in the DL-DWI protocol. Therefore, the observed benefits in scan time, image quality, and quantitative values represent the integrated effect of the acquisition adjustment and the deep learning reconstruction, not the isolated contribution of the latter. Future technical studies employing identical acquisition parameters with and without DL reconstruction are needed to precisely quantify the algorithm’s standalone impact. Fourth, the exceptionally high AUC values achieved must be interpreted in the context of our cohort’s composition. Our study population consisted predominantly of pathologically solid lesions that they were also 1 cm or greater (e.g., typical invasive carcinomas vs. fibroadenomas), which are generally easier to differentiate on imaging. Challenging borderline lesions, such as pure DCIS or complex benign entities, were underrepresented. This spectrum bias likely contributes to the near-perfect performance observed and means our results may not be directly generalizable to all clinical populations where diagnostic uncertainty is highest. The true test of this technique will be its performance in a prospective cohort enriched with such challenging cases. Fifth, the spatial resolution of our MRI acquisitions, particularly the 5 mm slice thickness used for both DWI and Synthetic MRI, must be considered. This resolution may contribute to partial volume averaging, especially for smaller lesions (<1 cm). This effect could influence the precision of quantitative parameter measurements, particularly for Synthetic MRI’s T1, T2, and PD maps, and may have limited our ability to detect more subtle differences or correlations. Future studies should implement thinner slices (4 mm or less) following European Society of Breast Imaging (EUSOBI) guidelines to improve quantitative accuracy and diagnostic performance, particularly for small and non-mass breast lesions. Sixth, the performance estimates for our models, while internally validated by standard ROC analysis, were derived without techniques such as bootstrapping or cross-validation. Consequently, the confidence intervals around the AUC and sensitivity/specificity estimates may be narrower than they would be with resampling methods, and the potential for optimistic bias due to overfitting cannot be ruled out. The reported near-perfect AUCs should therefore be interpreted as indicative of high potential within this specific cohort. This study reports discriminatory performance but does not define or validate a specific clinical decision threshold, nor does it report prevalence-dependent predictive values (PPV, NPV). Establishing a robust threshold and understanding the model’s performance across different pre-test probabilities are critical next steps for clinical translation and should be a primary focus of subsequent prospective trials.

## Conclusion

In conclusion, our proof of concept study demonstrates that deep learning-reconstructed DWI provides excellent diagnostic performance for differentiating benign and malignant breast lesions improved compared to the historical ADC for lesions 1 cm and larger and potential as a non-contrast technique. Although the combination with Synthetic MRI did not offer a statistically significant improvement in our cohort, the DL-DWI technique itself represents a significant advancement. Future studies with larger, prospective, and multi-center cohorts are essential to validate our findings and compare directly with CE-MRI. Furthermore, research correlating these advanced imaging parameters with specific histopathological subtypes and genetic markers will be crucial to unravel the biological underpinnings of the observed quantitative values, particularly the unexpected T1 findings, and to refine the optimal role for Synthetic MRI parameters in a DL-enhanced diagnostic workflow.

## Data Availability

The original contributions presented in the study are included in the article/supplementary material. Further inquiries can be directed to the corresponding author.
